# The Leech Nervous System: A Valuable Model to Study the Microglia Involvement in Regenerative Processes

**DOI:** 10.1155/2013/274019

**Published:** 2013-06-26

**Authors:** Françoise Le Marrec-Croq, Francesco Drago, Jacopo Vizioli, Pierre-Eric Sautière, Christophe Lefebvre

**Affiliations:** Lille 1 University, Fundamental and Applied Biology and Mass Spectrometry, FABMS-PRISM, Microglial Activation Group, IFR 147, SN3, 59655 Villeneuve d'Ascq Cedex, France

## Abstract

Microglia are intrinsic components of the central nervous system (CNS). During pathologies in mammals, inflammatory processes implicate the resident microglia and the infiltration of blood cells including macrophages. Functions of microglia appear to be complex as they exhibit both neuroprotective and neurotoxic effects during neuropathological conditions *in vivo* and *in vitro*. The medicinal leech *Hirudo medicinalis* is a well-known model in neurobiology due to its ability to naturally repair its CNS following injury. Considering the low infiltration of blood cells in this process, the leech CNS is studied to specify the activation mechanisms of only resident microglial cells. The microglia recruitment is known to be essential for the usual sprouting of injured axons and does not require any other glial cells. The present review will describe the questions which are addressed to understand the nerve repair. They will discuss the implication of leech factors in the microglial accumulation, the identification of nerve cells producing these molecules, and the study of different microglial subsets. Those questions aim to better understand the mechanisms of microglial cell recruitment and their crosstalk with damaged neurons. The study of this dialog is necessary to elucidate the balance of the inflammation leading to the leech CNS repair.

## 1. Introduction

Although long underestimated, microglia nowadays comprise an attractive target for accessing the diseased CNS. Microglial cells are regulators of tissue homeostasis in the adult central nervous system and readily participate in pathological processes, orchestrating tissue remodeling. In vertebrates, microglia are currently considered to be a kind of sensor in the brain because they respond to alterations in the brain and are activated by such changes [1]. Microglia constitute the first line of cellular defense mechanisms against central nervous system diseases [2], participating in the regulation of nonspecific inflammation as well as adaptive immune response [3]. That constitutes a very early stage in response to injury [4–6]. When the brain is injured or affected by diseases (e.g., degenerative, infectious, or autoimmune diseases), the resident ramified microglia morphologically transform into cells with retracted processes and enlarged cell bodies and increase in number at the affected site. Microglial cells with this particular form are generally referred to as “activated microglia” or “reactive microglia”.

The complexity of microglial responses is reinforced by the cell origin which is still controversial. In addition to the endogenous microglia which result from invasion processes in brain during early embryogenesis, studies showed that myeloid progenitors can penetrate into the brain even in normal adult mice to replace decaying microglial cells. In addition, phagocytes with morphological features of endogenous microglia might be derived from bone-marrow (BM) cells or from circulating monocytes during CNS diseases [7]. The complexity of microglia researches is also increased when we consider that the blood brain barrier is variably broken in these pathologies and that infiltrated cells might subsequently play a critical role in the disease.

Thus the microglial functions appear to be complex as they exhibit both neuroprotective and neurotoxic effects. In mammals, the CNS is supported and defended by two different macrophage populations: resident microglia and CNS-infiltrating macrophages. Importantly, despite the *in vitro* studies [9], morphological and histological *in vivo* analyses do not allow reliable discrimination of these two cell types because of common changes in form and marker staining. While the importance of ‘‘bone marrow-derived microglia” is highly questioned in neurodegenerative diseases, the understanding of the functional differences of infiltrating macrophages is a necessary prerequisite to elucidate successive steps, involving activated microglia, occurring in CNS pathologies [10].

## 2. The Leech CNS Mapping

**Figure 1:**
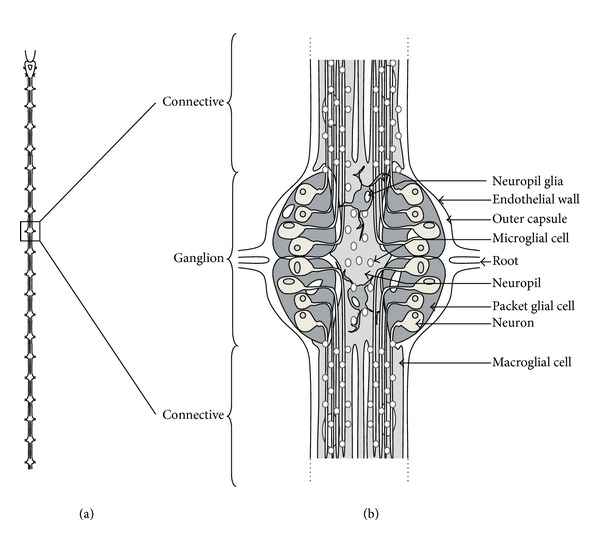
(a) Diagram of leech nervous system containing a head ganglion, 21 body ganglia, and 7 fused tail ganglia joined by connectives. (b) The dorsal view of the ganglion presents packet glial cells enveloping neuron cell bodies. The axonal processes passing through the neuropil are prolonged into connectives. The neuropil lies dorsomedially and contains two macroglial cells. Thousands of microglial cells are distributed in ganglia and connectives. The nervous system is enclosed in the outer capsule which is covered on the outside by a visceral layer of the endothelium (lining the ventral blood sinus). (Reprinted, with permission, from [8]).

*Hirudo medicinalis* is well studied in neurobiology because the CNS structure is tightly defined for decades [11]. Leech CNS is included in the ventral blood sinus and is constituted by head ganglion, 21 body ganglia, and 7 fused tail ganglia (Figure 1(a)). The ganglia are joined by connectives that consist of two large lateral bundles of nerve fibers and a thin medial connective called Faivre's nerve. Each segmental ganglion contains about 400 neurons and is linked to its neighbors by thousands of axons that form the connectives (Figure 1(b)). The other types of cells in leech ganglia are two connective glial cells that surround the axons, a neuropil giant glial cell and six packet-glial cells that ensheathe the cell bodies of neurons. In the adult CNS, microglial cells are small resident cells evenly distributed in the ganglia (more than 10000 for each one) and in the connectives (2000 for each one).

Since the observations of Retzius in 1891, the nerve cells in leech were studied for their morphological and, later, electrophysiological properties. The simple structure of the nervous system allowed studies about the specificity of synaptic connections. Thus individual neurons were functionally identified and mapped in each ganglion. Firstly, three groups of sensory neurons were discriminated and named touch (T), pressure (P), and nociceptive (N) cells [12]. Secondly, motor neurons (M) were identified [13]. The leech locomotive behavior—the swimming—has been studied following CNS lesions. Several electrophysiological and behavioral analyses allowed the *in vivo* studies of these leech processes. Some authors showed that individual sensory cells develop new synaptic connections with a high degree of specificity after a section, enabling to compensate the effects of the lesion by the restoration of the swimming [14, 15]. Synapse regeneration was demonstrated by *in vitro* experiments from isolated leech ganglia [16]. In addition, another identifiable cell, the single (S) interneuron, facilitated the study of specific connections between its axon and a single target cell. Each ganglion contains one S cell which is connected via its very long axon to that of the other interneuron in the neighboring ganglion. Of interest, unlike the mammalian brain, the leech CNS can be directly manipulated by intracellular injection of protease upon a synaptic target without any damage of the connected axon. In this context, if one S cell's axon is severed, the regenerative processes will form new synapses along its own distal stump leading to a new functional connection with its original target cell [17–19]. The mechanisms of nerve regeneration were progressively investigated by taking into account that other cell types are present close to the injured neurons. One type corresponds to the giant glial cells which are single in each connective and ensheathe axons in this region between adjacent ganglia. Some experiments permitting their individual destruction by intracellular injection of protease showed that, although their absence, the ability of a single damaged axon to reconnect with its specific target is not altered [20–22]. Thus these giant glial cells (macroglia) are not essential to engage the axonal sprouting. Other experiments have focused on another type of glial cells in the leech CNS, the microglia.

## 3. The Leech Microglia

Although microglial cells were described by a number of authors including Nissl [23], Robertson [24], and Alzheimer [25], they were first named “microglia” by Del Rio-Hortega in the leech CNS using his silver carbonate method [26, 27].

The different studies focusing on the leech microglia were justified by the structure of leech CNS which favors the study of resident microglia activation.

### 3.1. A First Observation: The Leech Microglia Migrate at the Lesion Site

**Figure 2:**
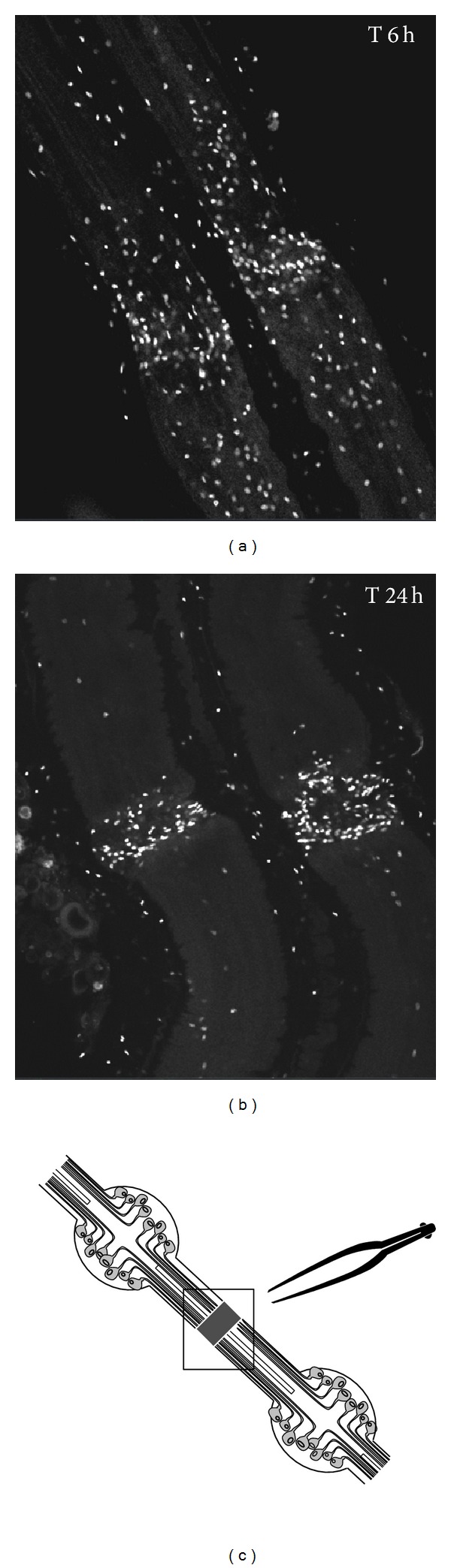
Observation of microglial cell accumulation from isolated and crushed segment of nerve cord maintained in tissue culture (see the diagram (c)). Microglial cell nuclei (white) were stained with Hoechst fluorescent dye to observe cell accumulation at the crush. (a) Six hours following a lesion of connectives, microglial cells progressively migrate toward the lesion site. (b) Later, 24 hours after the crush, microglial cells are mainly recruited to the lesion. This accumulation can be studied as much *in vivo *as *in vitro*.

**Figure 3:**
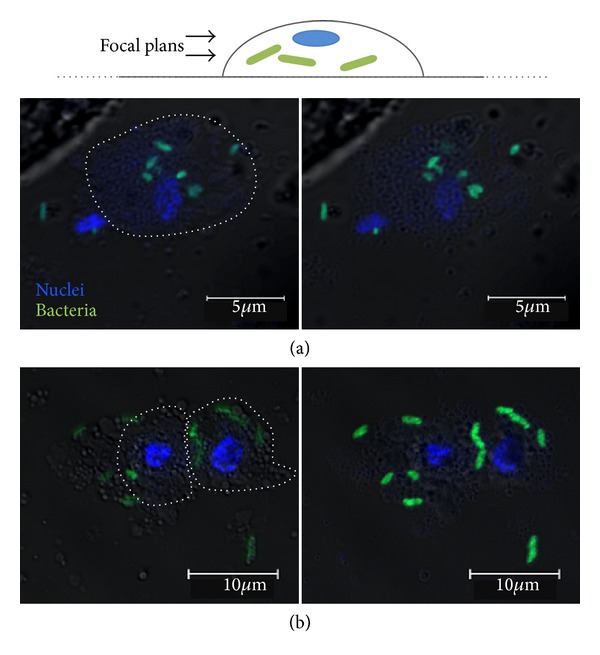
Phagocytosis activity of leech microglial cells. Following the dissection of leech nerve cord, microglial cells have been isolated and incubated in Ringer solution. Then heat-killed bacteria (*Aeromonas hydrophila*) have been labeled with fluorescein isothiocyanate (FITC) to be incubated during 1 minute, 10 minutes, 20 minutes, or 6 hours in respective identical plates containing microglial cell culture at room temperature. Finally, after fixation with 4% paraformaldehyde, the cytospinned cells have been incubated with a nuclear dye (10 *µ*g·mL^−1^ Hoechst 33342), washed in 0.1 M PBS, and mounted for analysis by confocal fluorescent microscopy (blue, nuclei; green, bacteria). Only 10 minutes (a) and 6 hours (b) conditions are presented in the figure.

Most observations of microglia in the leech were made by electron microscopy. Microglial cells have been evoked for the first time for the increase of their number after injury [28]. Morgese and collaborators then used two different histological techniques adapted for leech CNS tissue in order to see microglial cells in whole mount with the light microscope [29]. Weak silver carbonate, a classical stain for vertebrate microglia, allowed them to observe small glial cells similar to their vertebrate counterparts. The authors also used Feulgen's DNA specific staining for determining the distribution of leech microglial cells. Within 24 hours after the nerve cord is crushed, leech microglia aggregate to the site of injury [16]. Interestingly, some experiments were performed on isolated segment of nerve cord maintained in tissue culture (Figure 2). In such preparation, axon and synapse regeneration of the nerve cord has been shown to occur. In isolated connectives that were crushed, microglial cells clustered at the crush. Thus leech microglia can clearly respond to injury in the absence of blood [29]. These authors suggest that, thanks to the appearance of leech microglia following lesions, these cells could be phagocytic. They report an egress of glial cells from traumatized nervous tissue [29]. Observations in the leech suggested that these mobilized microglial cells may be engaged in clearing the damaged tissue of cellular debris. The present paper proposes simple experiments but unrealized on leech microglia to date and brings the evidence of the phagocytic activity of leech microglial cells (Figure 3). The analysis has been realized by using confocal microscopy which allows the observation inside the cells with successive focal plans. Only two focal plans are presented in the figure. The results show that leech microglial cells are able to rapidly phagocyte FITC-labeled bacteria (*Aeromonas hydrophila*). Though bacteria are not yet observed in cells after 1 minute, phagocytosis processes are significantly detectable following 10 minutes of incubation (Figure 3(a)). The mechanism is still observable after 20 minutes (data not shown) and 6 hours of incubation (Figure 3(b)) although the experiment does not reveal if it involves the same microglial cells or other additional ones in the time course of phagocytosis. That suggests that some microglial cells are able to eliminate debris in a short time. It is interesting to note that some experiments preincubating the cells in 1 mM ATP for 1 hour have been realized in similar conditions. The presence of that known microglial activator did not significantly modify the results in terms of kinetics or phagocyte number.

### 3.2. During the Response to a Lesion, the Leech Microglial Cells Change Their Morphology

**Figure 4:**
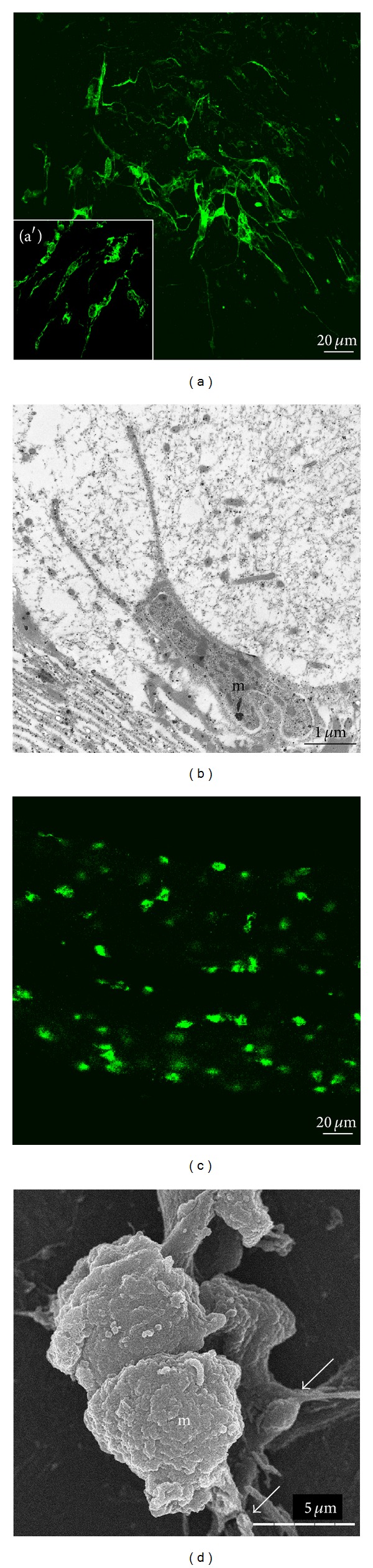
Transformation of resident microglia (m) into activated cells allowing their migration to the injury site. (a) and (c) Immunohistochemical staining using anti-gliarin antibody in ramified microglial cells from connectives (a), ramified microglial cells from ganglia (a′), and rounded microglial cells during their recruitment in connectives (c). (b) Transmission electron microscopy of an elongated microglial cell (m) with thin pseudopods in a naive ganglion. (d) Scanning electron microscopy of activated microglial cells accumulated at the lesions. Once recruited, the cells do progressively develop new ramifications (arrows). (Reprinted, with permission, from [31]).

Conformational changes have been described as necessary to allow natural functions of microglial cells [30]. Following a crush in the connectives, microglial cells migrate at the lesion. As previously described in mammalian microglia, this mobility has recently been associated to a transformation from stellate to rounded shape (Figure 4) [31]. The progressive amoeboid feature is comparable to those indicatives of the process of activation in the mammalian immunocytes. Once recruited, the microglial cells interact with damaged neurons allowing neurite outgrowth. Some observations showed that microglial cells need to be ramified to crosstalk with neuritis [32]. Indeed, leech microglial cells can be isolated from a freshly dissected nerve cord. Then microglial cells may be cultured *in vitro*. By using concanavalin-A (Con-A) as a coating, a high percentage of cells are still rounded. When cells are cultured on Con-A in the presence of injured CNS factors, the number of rounded cells decreases to 48% meaning that the other ones are able to develop processes (intermediate form) [30]. This change of morphology allowed more growth of neurites across microglial cells. Thus some diffusible substances from injured CNS tissue are responsible of the morphological transition of the microglial cells. When microglial cells are plated on laminin, they are mainly spindle shaped and interact with growing neurites.

Thus in this context, laminin, a glycoprotein molecule from CNS extracellular matrix, appeared within the connectives in the region of neurite outgrowth suggesting that it may promote axonal growth in the CNS of the animal as in culture [32]. Leech laminin could provide a favourable substrate that induces efficient neurite outgrowth of some neurons in culture, but the cells which produce laminin at the crush site have to be identified. Even following the ablation of giant glial cells, microglial cells were concentrated at the sites of new laminin appearance and axon sprouting. This observation suggests that microglial cells may be responsible for the appearance of new laminin molecules contributing to sprouting [30, 33].

As a result, diffusible factors including laminin could be necessary to condition the functional features of microglia in their crosstalk with damaged neurons at the lesion site.

### 3.3. Dynamical Studies of Microglia Recruitment: Structural Advantages

The previous data show that leech microglial cells have similar properties with mammalian counterparts through their mobility, their phagocytic activity, and their morphological changes during activation processes. Interesting features of the leech nerve cord structure can explain the story of the leech neurobiology. Because the central nervous system (CNS) is organized as a tubular nerve cord with adjacent ganglia joined by connectives, it facilitates the study of the nerve cells. The neuronal cell bodies are mapped in each ganglion [34] processing their axon into the connectives. It firstly results in that mechanisms following nervous system injury can be analyzed at the single cell level [19]. Since the microglia accumulation at the lesion site is considered as an important and rapid recruitment to engage axonal sprouting, it secondly results in that this cell movement can be dynamically studied by *in vivo* imaging of the connectives. Importantly, microglia cannot be confused with other cells because there are neither astrocytes nor oligodendrocytes in leech CNS. Finally, the absence of blood vessels within the central nervous system does structurally simplify the separation between CNS and blood. Thus the leech inflammatory response may be explored from the sinus-free CNS. Anyway, the very low infiltration of blood cells which have been observed in injured CNS—even surrounded by the blood sinus—highlights the importance of the resident microglia at the lesions [29, 35]. As a result, leech microglial cells may be studied for their contribution to the CNS repair without any infiltrating blood cell contribution. Since video microscopy is possible to observe Hoechst-stained microglial cells moving to the lesion site, some studies—using isolated segment of nerve cord maintained in tissue culture—showed the migration kinetics of these cells into the leech connectives [36]. Thus, microglia start to move within the first minutes after the injury and accumulate at the lesion site within 2 hours. During this recruitment, only a fraction (less than 50%) is able to move at any time at speeds up to 7 *µ*m per minute [36].

Because the microglia accumulation at the site of lesion is known as a rapid and important process for the usual sprouting of injured axons in leech *Hirudo medicinalis*, the subsequent studies aimed to identify the molecular processes responsible of the microglial recruitment following lesions in the leech CNS.

### 3.4. Molecular Mechanisms Involved in Microglial Migration

#### 3.4.1. Nitric Oxide and Purines

A first explanation of molecular mechanisms came from the endothelial nitric oxide synthase (eNOS) activity in the injury site as a very early response suggesting that NO is involved in the microglial recruitment at the lesions [37]. Moreover, some experiments using NO inhibitors resulted in a decrease of microglial cell accumulation suggesting that NO contributes to microglia chemotaxis [38]. Importantly, the use of spermine NONOate (SPNO) as an NO donor at the lesions also reversibly blocked the accumulation of microglia [38]. These data suggest that NO could attract the microglial cells at low concentrations (distant from the lesion site) promoting their movement and also serves as a stop signal for migrating microglia at a high concentration (at the crush). Further studies using NO microsensor demonstrated the rapid efflux of NO at the lesions controlling the microglial accumulation [39]. Nitric oxide is also known to activate a soluble guanylate cyclase (sGC) leading to cyclic GMP (cGMP) production. Immunohistochemical experiments using anti-eNOS and anti-cGMP antibodies showed a similar increase of eNOS and cGMP at the lesion site. When methylene blue is used as a soluble guanylate cyclase (sGC) inhibitor, the cGMP immunoreactivity is abolished which is correlated with the inhibition the microglial accumulation at the lesion [40]. Actually, the decrease of cell number at the lesion results from the misdirection of moving cells. The use of NO scavenger (cPTIO) confirmed the involvement of NO in a cGMP-dependent signaling pathway by decreasing the cGMP immunoreactivity [41]. Importantly, the NO scavenger did not reduce the microglial cells movement but altered their directionality. In mammals, NO/cGMP signaling pathway activates a protein kinase G, called PRKG1 (alias cGK1 or cGKI), which phosphorylates serines and threonines on many proteins leading to the modulation of cellular calcium. Proteins that are phosphorylated by PRKG1 are then involved in the CNS to promote, for example, the axon guidance [42]. Although the functional involvement of PRKG1-related molecule has not been so far specified in NO-dependent activities in leech CNS, we have recently identified at least two molecules presenting high similarities with mammalian and invertebrate PRKG1 homologs. Further studies would focus on their cell localization in order to better understand NO effects on target cells. Thus, NO is the first diffusible molecule identified in the leech CNS that is essential to organize the microglial movement toward the lesion. Other molecules are able to engage the leech microglial movement. Indeed ATP, ADP, and UTP have been investigated for their ability to activate the microglial accumulation [41, 43]. Though ATP could be released via innexins from glia to activate microglial cells [44, 45], further studies are still necessary to identify the leech receptors for such chemotactic molecules. The use of reactive blue 2 (RB2) as an antagonist of purinergic receptors slowed the migration (but did not misdirect the cells in movement) suggesting the existence of such a receptor [41]. The analysis of the leech genome since these last two years hypothesizes the presence of purinergic receptors in leech CNS but has to specify their functional features (unpublished data).

By taking into consideration the advantage of the crushing on an isolated segment of nerve cord, Ngu and colleagues [43] used specific inhibitors for ATP- or NO-dependent activities in order to reduce the microglial cell accumulation (by slowing or disorientating the cells) and then study the consequences on the growth of severed axons. To measure this correlation, sensory neurons were injected with biocytin to label their sprouting after lesioning. Remarkably, in spite of the lesion, when microglial accumulation has been reduced by inhibitors, the authors observed a significant reduction in total sprout lengths on damaged neurons. Consequently, microglia are shown to be essential for the usual sprouting of injured axons [43].

ATP and NO are diffusible molecules presumably released within the first minutes following CNS lesions. Because we know that the microglial accumulation is a rapid but progressive process, other studies have been directed to identify the released factors which are chemotactic for microglial cells in the time course of the accumulation.

In order to characterize potential chemotactic factors involved in the recruitment of the microglial cell population, some studies have taken advantage of the establishment of databases such as Expressed Sequence Tag (EST) library from the leech CNS and the leech genome [46]. According to the sequence homologies with known chemotactic factors and the presence of conserved domains, some products have been functionally investigated.

#### 3.4.2. *Hm*EMAPII

A molecule homologous to the human complex p43/endothelial monocyte-activating polypeptide II (EMAPII) was recently characterized in the leech CNS [47]. In mammals, the p43 precursor is known to be released after processing the cytokine EMAPII which is suggested to be a marker of microglial cell reactivity [48–50]. EMAPII is highly produced in activated microglia of injured brain suggesting its involvement in inflammatory and neurodegenerative pathologies [51, 52].

Human EMAPII has been shown to exert a chemoattractant effect on both leech and human microglial cells. Although it is considered important in microglial activation, its chemotactic function has been described for the first time in the leech microglial [47]. The leech gene coding for *Hm*EMAPII (for *H. medicinalis*) results from a signaling pathway associated with a recently characterized leech TLR in the CNS, called *Hm*TLR1 [47, 53]. Since the CXCR3 molecule is the natural receptor for human EMAPII, chemotaxis assays on leech microglia have been performed using either anti-CXCR3 antibody or IP-10, an agonist of CXCR3. The results showed a competitive effect allowing the reduction of EMAPII-induced microglia migration, suggesting the existence of a CXCR3-related molecule in the leech [47].

#### 3.4.3. *Hm*IL-16

The importance of interleukin-16(IL-16)-related molecule has been described in the microglia recruitment of the leech CNS [31]. In mammals, IL-16 is a proinflammatory cytokine originally identified as a lymphocyte chemoattractant factor [54, 55] and produced by numerous cells including lymphocytes and microglia [56]. The mature IL-16 corresponds to C-terminal peptide of a precursor which subsequently acts by forming bioactive multimers [57]. Indeed, the homotetramerization of human IL-16 was reported as the optimal biological activity [58]. In human brain, IL-16 is constitutively expressed by a microglia subpopulation and may attract CD4+ lymphocytes across the blood brain barrier under pathological conditions [59]. A paracrine role of IL-16 was also demonstrated in inflammation following cerebral ischemia [60]. IL-16 may be implicated in some neurodegenerative diseases such as multiple sclerosis (MS) where enhancement of IL-16 production suggests a role in the regulation of inflammation in axonal damages [60–63].

In the leech, a new molecule, designated *Hm*IL-16, is produced in naïve neurons but rapidly induced following a lesion and transported along the axonal processes to promote the recruitment of microglial cells to the injured axons [31]. *Hm*IL-16 can be produced in microglia at least 72 hours after the injury. Thus microglial cells might first be activated by neuronal *Hm*IL-16 which is released from damaged neurons. Then these recruited microglial cells could release their own cytokine to maintain cell accumulation at the lesion. *Hm*IL16 possesses functional homologies with its human counterpart, by exerting chemotactic activity as it has been similarly observed using human IL-16 on leech microglial cells. By using *in vitro* chemotaxis assay, preincubation of microglial cells either with an anti-human IL-16 antibody or with anti-*Hm*IL-16 antibody significantly reduced microglia migration induced by injured leech CNS-conditioned medium. Remarkably, functional similarities have been demonstrated by the ability of *Hm*IL-16 to promote human CD4+ T cell migration [31].

The fact that *Hm*IL-16 has no effect on CD8+ cell migration further indicates a similar relationship with CD4 as described for human IL-16. Interestingly, the activity of *Hm*IL-16 contained in the conditioned medium has been inhibited by using a soluble CD4 as competitor for the membrane CD4. Although a CD4-related protein has not been identified in leech to date, these results would strongly suggest its presence. Indeed, these results indicate that *Hm*IL-16 might act via a CD4-related molecule on the leech microglial cells as reported for human microglia [59]. Preliminary results using the human anti-CD4 antibody allowed the reduction of human IL-16-induced leech microglia migration (unpublished data). Finally, the same antibody evidenced the presence of an immune-reactive product in leech CNS and in leech protein extracts eluted on affinity column using IL-16-coated beads (unpublished data). The following study is still in progress and would presumably lead to the characterization of such a related molecule in the leech.

#### 3.4.4. *Hm*C1q

A molecule homologous to vertebrate C1q has been recently characterized in the leech CNS [8]. In vertebrates, members of C1q family, C1qTNF proteins are involved in triggering and regulation of various inflammatory reactions [64]. Among the mediators expressed by microglial cells and neurons, C1q seems to be a key molecule in neuroinflammatory diseases, that is, systemic lupus erythematosus (SLE) [65, 66]. It is also involved in various neurodegenerative pathologies as Alzheimer disease [67, 68]. In addition, C1q is known to drive microglial activation [69]. Beside the nervous system, the chemotactic properties of C1q have been demonstrated for immune cells [70–72]. Finally, their migration is mediated through recognition of both gC1qR and cC1qR [73].

In the leech, *Hm*C1q is produced at least in neurons and glial cells. In chemotaxis assays, leech microglial cells were firstly demonstrated to respond to recombinant human C1q [8]. Then, while the use of injured leech CNS-conditioned medium, as *in vitro *chemoattractant, exhibited a significant dose-dependent chemotaxis on leech microglia, its effect was reduced when cells were incubated with anti-*Hm*C1q antibodies. Thus, *Hm*C1q released in the culture medium contributes through its chemotactic effect to the microglial recruitment. Its activity has been correlated to nitric oxide since the NO scavenger (cPTIO) partially abolished the *Hm*C1q effect on the leech microglia recruitment. This result highlights that the C1q effect is related to the presence of NO which plays a key role in leech microglia recruitment as discussed above. *Hm*C1q activity were reduced when microglia were preincubated with signaling pathway inhibitors such as pertussis toxin or wortmannin suggesting the potential involvement of G-proteins and phosphoinositide 3-kinases in the pathway of the C1q-induced signal for chemotaxis [8]. Remarkably, again in chemotaxis assays, when the cells have been preincubated with anti-human gC1qR antibody, the *Hm*C1q effect has been significantly reduced. That result suggests the involvement of gC1qR-related molecule in C1q-mediated migration in leech.

**Figure 5:**
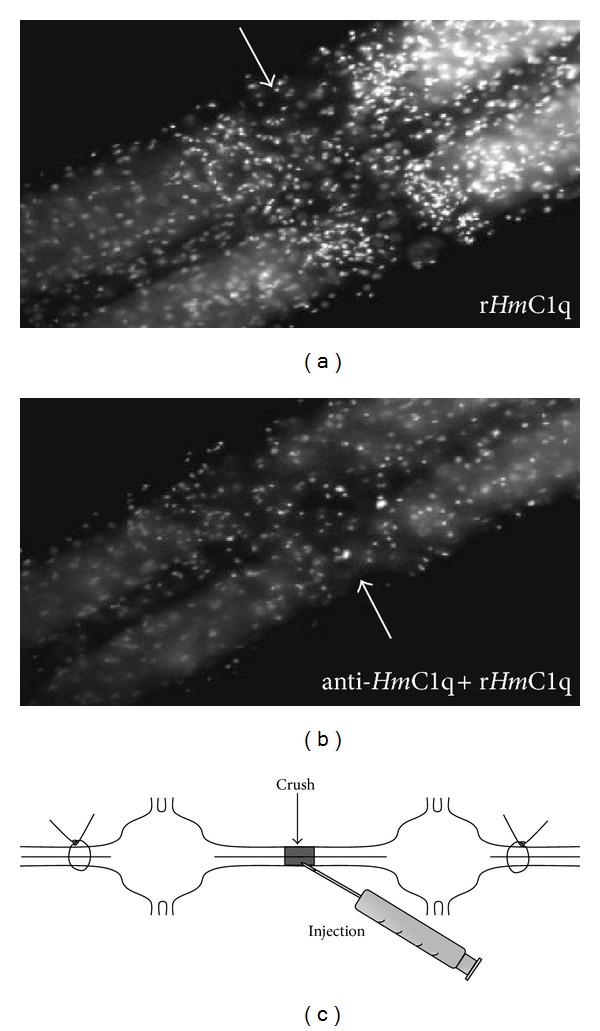
Chemotactic effect of recombinant *Hm*C1q on leech microglia in isolated and crushed segment of nerve cord maintained in tissue culture (see the diagram (c)). (a) Hoechst-dyed microglial cells strongly accumulated 4 hours after injection of r*Hm*C1q (arrows). (b) When tissues have been injected with polyclonal anti-*Hm*C1q antibody, the chemotactic activity of r*Hm*C1q has not been observed (arrows). To note, the corresponding preimmune serum did not exert any neutralizing effect (data not shown). (Reprinted, with permission, from [74]).

The production of the recombinant form of *Hm*C1q permitted specifying its importance in the microglia migration into the connectives [74]. As discussed above, *ex vivo *experiments can be performed on the isolated segment of nerve cord maintained in tissue culture. In such preparation, it is interesting to note that microglial cells are still reactive and recruited in crushed connectives. This original feature allows the injection of chemotactic factor with or without inhibitors to enhance and/or regulate the Hoechst-dyed cell accumulation. By taking advantage of this natural ability, recombinant *Hm*C1q exhibited a strong chemotactic effect on microglial cells which has been abolished when recombinant *Hm*C1q is simultaneously injected with anti-*Hm*C1q antibody (Figure 5).

**Figure 6:**
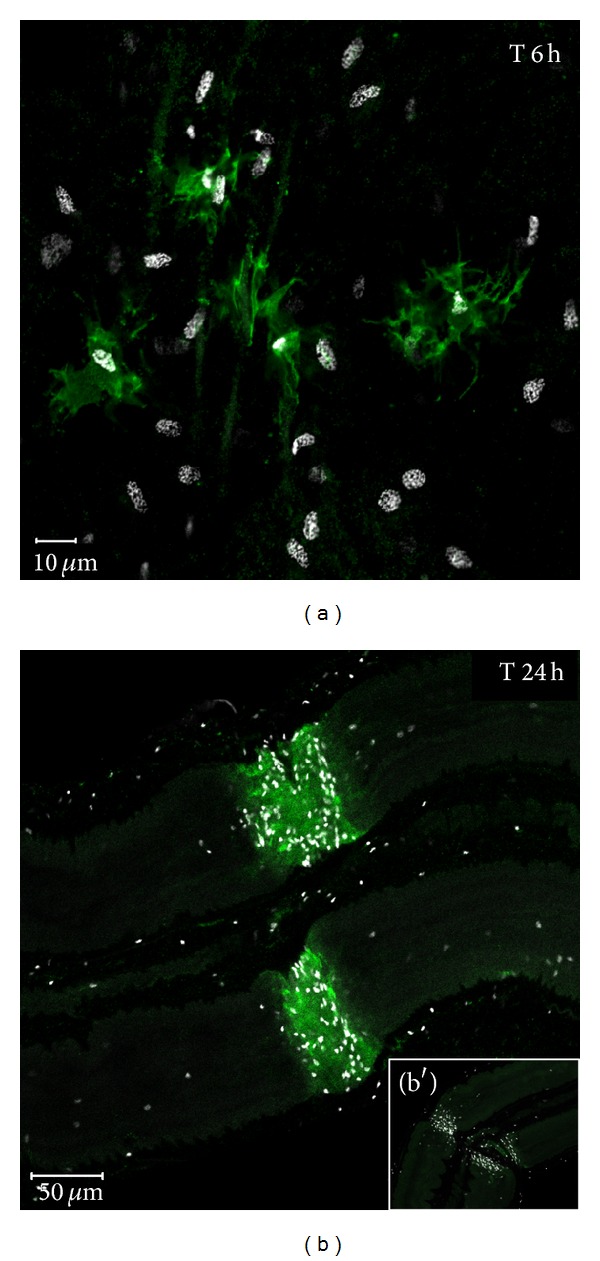
Immunostaining of leech central nervous system (CNS) using rabbit polyclonal anti-human C1qBP antibodies (green). Microglial cell nuclei (white) were stained with Hoechst fluorescent dye to observe cell migration. (a) High magnification image of the injury site after 6 hours. Since all microglia nuclei are shown by Hoechst counterstaining (white), images demonstrate that the anti-C1qBP immunostaining selectively enhances some microglial cells. (b) 24 hours following injury, the number of microglial cells is much higher at the lesion site, and stronger positive immunostaining is observed. (b′) No immunostaining was observed using secondary antibodies alone as negative controls. (Reprinted, with permission, from [74]).

Then, the existence of a receptor for *Hm*C1q (called *Hm*gC1qR or *Hm*C1qBP) in the leech CNS has been demonstrated following its molecular characterization from leech databases [74]. The involvement of *Hm*C1qBP in *Hm*C1q-dependent chemotaxis has been definitely ascertained showing the inhibition of *Hm*C1q effect on *in vitro *leech microglia migration by using anti-C1qBP antibodies. Finally, affinity purification and flow cytometry experiments showed the interaction between *Hm*C1q and *Hm*C1qBP molecules. In mammals, such interaction was identified in dendritic cells but has never been shown in nerve cells [73]. In addition, the interaction between *Hm*C1q and its receptor is the first evidence of the molecular reactivity of microglial cells during their accumulation. Interestingly, that receptor has been localized only in a portion of microglial cells (Figure 6). Thus, a well-defined subpopulation of Hoechst-dyed microglial cells recruited at the crush is *Hm*C1qBP positive and reactive to *Hm*C1q demonstrating that microglia cannot be considered as a whole reactive population.

We presently know the involvement of different chemotactic factors in the microglia recruitment to the lesion. Additional studies might evaluate the activity of their respective recombinant form in a time course and a dose-dependent manner on microglia recruitment. That question is crucial to better understand the hierarchy of chemoattractants and the chronological responses delivered to damaged neurons.

It is also necessary to continue the identification of the molecules involved in microglial activation to fully understand their functional relevance. A recent study highlighted the importance of endocannabinoids, namely, N-arachidonylethanolamide (AEA) and 2-arachidonyl glycerol (2-AG), in the balance of NO and ATP release contributing to the microglia accumulation at the crush [75]. In addition to chemotactic factors, the identification of molecules regulating the balance of inflammatory response and activating the microglia/neuron crosstalk would help to understand the microglial functions in the leech CNS repair.

## 4. Role of Activated Microglia and Crosstalk with Damaged Neurons

The existence of different microglial subpopulations depending on their activating factors is strongly suggested in the leech CNS. Firstly, microglial cells have to be discriminated as a reactive population and nonreactive population. Indeed, injured leech CNS-conditioned medium is able to attract only a part of a whole microglia population.

The involvement of several activating and migrating signals acting on different subsets of microglial cells at the lesion site could be taken into account as suggested for mammals [10, 76]. The leech receptors for every chemoattractant of interest will allow the discrimination of microglial cell population depending on their reactivity to respective chemoattractant. Beside the existence of different activation molecules, it is necessary to understand the functions of recruited microglial cells at the end of damaged axons. Because microglial cells can be isolated from injured CNS, they are maintained in culture in order to be stimulated by respective chemotactic factors (C1q, EMAPII, or IL-16). The molecules from activated microglia which are secreted in the medium can be identified by using potent proteomic tools (nano-liquid chromatography coupled to orbitrap MS analysis) and by analyzing the leech genome. When compared to each other and with unstimulated microglia secretome, the analyses of differential secretomes already revealed products of interest depending on the activation processes (unpublished data). The identification of such products would help to understand the functions of only resident microglia at the lesion. Indeed, unlike mammals, the microglial accumulation in injured leech CNS does not involve any infiltrated blood cells. In mammals, it is not easy to discriminate the role of infiltrating macrophages sharing the same molecular markers as the resident microglia. Moreover, more and more studies show that infiltrated macrophages can exert distinct functions in the balance of inflammation depending on their polarization between M1 (pro-inflammatory) and M2 (neuroprotective) phenotypes [77]. Because nerve repair spontaneously occurs in leech nerve cord, we can hypothesize the existence of a particular neuroprotective microglial phenotype. In order to achieve the functional properties of activated microglial cells at the lesion, each secretome would be tested for its capacity to outgrow damaged neurons *in vitro*. By taking into consideration the advantage of the leech CNS facilities, notably the *in vivo* study of the axonal sprouting [16, 17], the injection of regenerative secretomes directly on crushed connectives would be possible in order to measure the time course of regenerative processes. Actually, some links might exist between the leech neuroinflammation and mammalian degenerative diseases since we observed that leech molecules interact with human immune cells [31] and that human factors act on leech microglia [8, 31, 47]. The different factors which were described in the leech CNS are more and more studied in mammals in neuroinflammatory processes as, for example, the human C1q which is a microglia activator [69]. These elements led us to investigate a possible link between the fundamental research and clinical application by using leech molecules in the rat spinal cord injury (SCI) model. The potential neuroprotective roles of leech CNS-secreted molecules have been assessed on rat SCI model in collaboration with Pr. Cizkova (Slovak Academy of Sciences, Kosice, Slovakia). Preliminary results using leech CNS-conditioned medium on dorsal root ganglion (DRG) neurons from adult rat show a rapid and significant neurite outgrowth (unpublished data).

## 5. Conclusion

Unlike vertebrates, some invertebrate animals can fully repair their central nervous system (CNS) following an injury. The CNS of medicinal leech is efficiently and functionally regenerated following lesions [14, 15]. In this context, the implication of microglia is a key step to engage an adapted response leading to the axonal sprouting. To summarize, in contrast to mammalian CNS, distinctions between the role of resident microglia and that of blood infiltrating macrophages—which contribute to neuroinflammatory mechanisms in mammals—are possible in leech CNS. Considering its easy manipulation and structural context, the leech CNS allows the *in vivo* and *in vitro *studies of activated microglial cells. This valuable model would offer interesting molecular and cellular bases to evaluate the consequences of microglia involvement in regenerative processes.
